# Crystal structure of 1*H*-imidazole-1-methanol

**DOI:** 10.1107/S2056989022002614

**Published:** 2022-03-10

**Authors:** Megan M. Wysocki, Gorana Puzovic, Keith L. Dowell, Eric W. Reinheimer, Deidra L. Gerlach

**Affiliations:** a Department of Chemistry and Biochemistry, University of Wisconsin-Eau Claire, 101 Roosevelt Ave, Eau Claire, WI, 54702, USA; b Rigaku Americas Corporation, 9009 New Trails Drive, The Woodlands, TX, 77381, USA

**Keywords:** crystal structure, hydrogen bonding, substituted imidazole

## Abstract

The synthesis and the crystal structure of 1*H*-imidazole-1-methanol, C_4_H_6_N_2_O, are described. This compound comprises an imidazole ring with a methanol group attached at the 1-position affording an imine nitro­gen atom able to receive a hydrogen bond and an alcohol group able to donate to a hydrogen bond. This imidazole methanol crystallizes with monoclinic (*P*2_1_/*n*) symmetry with three symmetry-unique mol­ecules. These three mol­ecules are connected *via* O—H⋯N hydrogen bonding in a head-to-tail configuration to form independent three-membered macrocycles.

## Abstract 2

The crystal structure of methanol imidazole featuring a tri-mol­ecule hydrogen-bonded macrocycle is described.

## Chemical context

Imidazole structures have occupied a unique position in heterocyclic chemistry as a synthetic precursor to imidazole salts ultimately for the formation of *N*-heterocyclic carbenes (NHCs) (Jahnke & Hahn, 2016 ▸). Nitro­gen heterocyclic carbenes (NHC) were first established by Skell in the 1950s and have been additionally developed by Fischer and his students who introduced carbenes into organic and inorganic chemistry in 1964 (Herrmann & Köcher, 1997 ▸). NHCs have proven to be excellent ligands in metal-based catalysis (Enders *et al.*, 2007 ▸). 1*H*-Imidazole-1-methanol, the mol­ecule highlighted in this article, is a precursor to form the NHC moiety of a larger tridentate ligand being developed. Although this mol­ecule’s synthesis and characterization has previously been reported by DeBerardinis *et al.* (2010 ▸), the crystal structure and structural properties have not. Conveniently, crystalline 1*H*-imidazole-1-methanol is directly obtained from the reaction of imidazole and paraformaldehyde. 
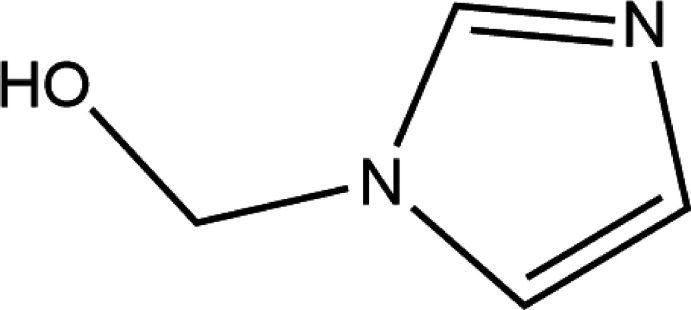


## Structural commentary

**Figure 1:**
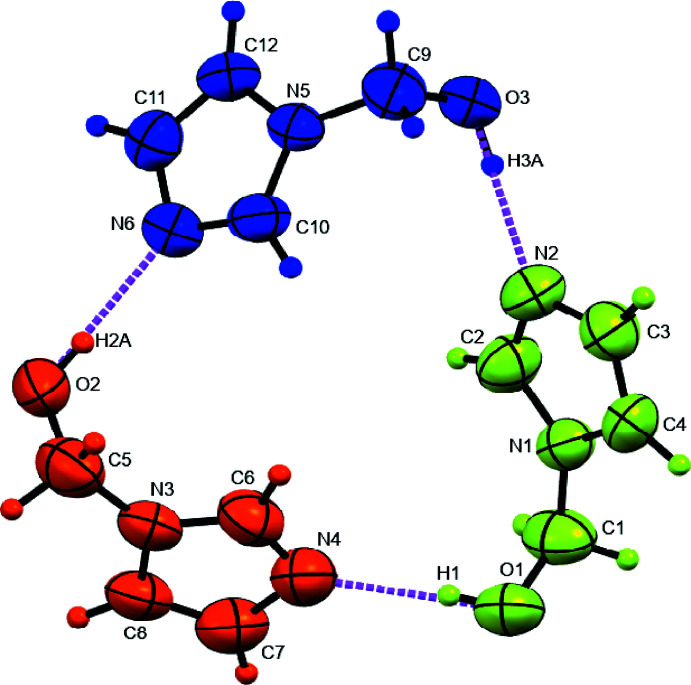
The asymmetric unit of 1*H*-imidazole-1-methanol represented with displacement ellipsoids at the 50% probability level. Inter­molecular hydrogen bonds are colored magenta with the three mol­ecules colored and numbered for quick identity in the discussion. The coloring and numbering correspond to the numbering of the oxygen atom for each as follows: (**1**) light green, (**2**) orange, and (**3**) blue (if displayed or printed in grayscale: (**1**) lightest gray, (**2**) medium gray, and (**3**) darkest gray).

The title compound crystallizes in the space group *P*2_1_/*n*, with the asymmetric unit of the cell containing three unique 1*H*-imidazole-1-methanol complexes (*Z*′ = 3), which are connected *via* head-to-tail hydrogen bonding. While each mol­ecule is identical in formula and connectivity, it is the torsion angle of the methanol substituent with respect to the imidazole ring that distinguishes the unique symmetry of the three mol­ecules. These deviations of torsion angle are isolated conformations essential to achieve the three-mol­ecule hydrogen-bonded macrocycle as seen in Fig. 1 ▸. Color as well as the label of the lone oxygen atom of each mol­ecule is used to distinguish the three symmetry-unique mol­ecules that create the macrocycle through hydrogen bonding. Note the mol­ecules are labeled as **1**, **2**, and **3** to correspond with O1, O2, and O3 with the respective colors of light green, orange, and blue. The torsion angle is measured from the oxygen, methyl­ene carbon, imidazole nitro­gen, and atom C2 of the imidazole ring (the carbon between the two nitro­gen atoms of the ring). The measured torsion angles are −102.0 (2), −80.2 (2), and 90.6 (2)° for mol­ecules **1** (O1—C1—N1—C2), **2** (O2—C5—N3—C6), and **3** (O3—C9—N5—C10), respectively. Note that the blue mol­ecule **3** has the methanol substituent rotated positively while the others are negative to achieve the geometry necessary to complete the hydrogen-bonded superstructure. While the isolated torsion angles observed are imparted by the hydrogen bonding, none of the bond angles, bond lengths, or torsion angles throughout each of the three asymmetric mol­ecules is unusual when compared with structures deposited with the Cambridge Crystallographic Database accessed *via Mogul* (Bruno *et al.*, 2004 ▸).

## Supra­molecular features

**Figure 2:**
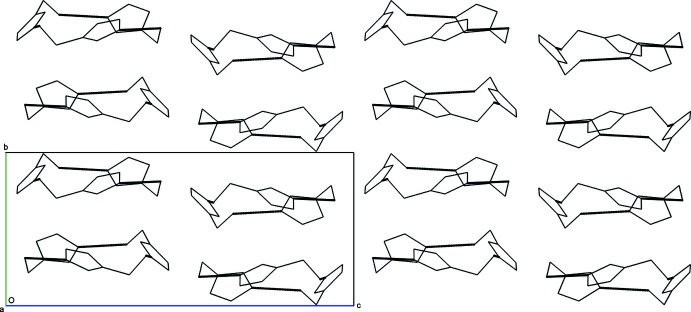
The packing arrangement of the three-mol­ecule supermolecule with hydrogen bonds represented as dashed lines as viewed along the *a* axis of the unit cell. The approximate diameter of each three-member supermolecule is 9–9.5 Å with a height of about 2.6 Å.

**Figure 3:**
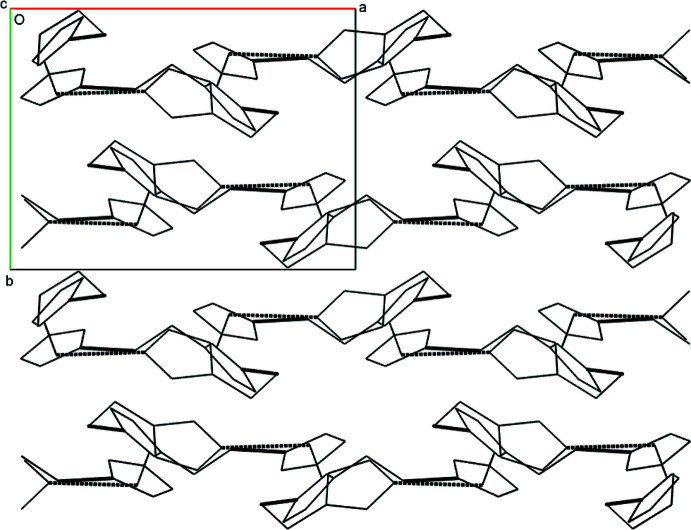
The packing arrangement of the three-mol­ecule supermolecule with hydrogen bonds represented as dashed lines as viewed along the *c* axis of the unit cell. Observed at this angle is the π-stacking of mol­ecule **1** with itself across an inversion center.

The hydrogen bonds (Table 1 ▸) inter­connecting mol­ecule **1** to mol­ecule **2** to mol­ecule **3** back to mol­ecule **1** are within the range of typical hydrogen-bond lengths for a hydroxyl oxygen donor to an imidazole nitro­gen acceptor. These lengths are 2.741 (2) Å for O1—H1⋯N4 (light green to orange), 2.753 (2) Å for O2—H2*A*⋯N6 (orange to blue), and 2.715 (2) Å for O3—H3*A*⋯N2 (blue to light green). The resulting triangular supra­molecule has a rough diameter of 9 to 9.5 Å and height of about 2.6 Å (Fig. 2 ▸). These triangular disks lie within the *ac* plane and offset stack along *b via* a twofold screw axis (Fig. 3 ▸). Views along each axis of the unit cell highlight the crystal packing of the supermolecule seen in Figs. 2 ▸, 3 ▸ and 4 ▸.

While the hydrogen-bonded supermolecule dominates the crystal packing, cofacial π-stacking occurs between mol­ecule **1** and itself about an inversion center at (0.5, 0.5, 0.5). A centroid was first calculated for the imidazole ring of mol­ecule **1** using N1, C2, N2, C3, and C4; the distance from centroid-to-centroid across the inversion center is 3.7219 (3) Å and is within typical values for π-stacked systems (Dance, 2003 ▸). It is noted that there is considerable void space when the crystal packing is viewed with typical van der Waals radii in spacefill mode utilizing any software; it can be hypothesized that this significant void space contributes to the low melting point of this compound.

## Database survey

The Cambridge Structural Database (CSD, version 5.42, update of 11/20; Groom *et al.*, 2016 ▸) contains many unique and different imidazole structures. A search for C_4_H_6_N_2_O in the database provided eight hits, none of which were the same complex or space group as the crystal structure formed. Of the structures found in the CSD, the most similar compound is 1*H*-imidazol-4-yl­methanol (AGAWIQ; Sanders *et al.*, 2013 ▸). While the other mol­ecules found by this search for the same empirical formula and contain a five-membered *N*-heterocycle (pyrazole, oxazole, and pyrrole) only AGAWIQ is also a substituted imidazole with a methyl­ene linker to a possible hydrogen bond acceptor/donor. Because the methanol substituent for this mol­ecule is at C4 of the imidazole ring, the same cyclic supermolecule does not form for this mol­ecule even though hydrogen bonding is exhibited. One mol­ecule forms the asymmetric unit for AGAWIQ with the hydroxyl oxygen being both a hydrogen-bond donor and acceptor to two different adjacent imidazole nitro­gen atoms behaving as acceptor, non-protonated N, and donor, protonated N, respectively. The title mol­ecule is not capable of this highly inter­connected hydrogen-bonding network due to the methanol substitution being at N1 of the imidazole ring, removing the possibility of a nitro­gen hydrogen-bond donor. The remaining seven hits included 3-methyl-3-pyrazolin-5-one (MPYAZO10, MPYAZO11; De Camp & Stewart, 1971 ▸; Zhang *et al.*, 2004 ▸), 3-methyl-1,2-oxazol-5-amine and 5-methyl-1,2-oxazol-3-amine (NOSZAZ and NOSZED; Morozova, *et al.*, 2019 ▸), 3-methyl-*N*-hydrox-pyrazole (REHKOE; Reuther and Baus, 1995 ▸), and *N*-nitro­sopyrroline (UCONIJ, UCONIJ01; Ohwada *et al.*, 2001 ▸; Marsh & Clemente, 2007 ▸).

## Synthesis and crystallization

The synthesis of 1*H*-imidazole-1-methanol was adapted from DeBerardinis *et al.* (2010 ▸). Under argon, imidazole (11.38 g, 167.2 mmol) was added to an ice-cold mixture of para­formaldehyde (5.01 g, 167 mmol) and 1,4-dioxane (45 mL, degassed), in a two-neck round-bottom flask equipped with a stir bar, condenser, and a vacuum adapter. Once all the reagents were added, the reaction mixture was removed from the ice bath, brought to room temperature, and stirred for an additional 2 h. The reaction was heated at 334 K overnight with stirring for 12 h. The mixture was cooled to room temperature and the 1,4-dioxane was removed under reduced pressure. The clear and colorless liquid collected was stored at 255 K. The final crystallized product, 1*H*-imidazole-1-methanol, was obtained as a white moist solid (11.6166g, 70.9%) and stored at 277 K. The resulting product was analyzed at room temperature on a Bruker Avance II 400 MHz NMR spectrometer for ^1^H NMR, a Bruker Alpha II ATR for FT–IR, and Electrothermal Meltemp for melting point. ^1^H NMR (400 MHz, CDCl_3_, δ in ppm): 5.40 (*s*, 2H), 6.93 (*s*, Im), 7.08 (*s*, 1H), 7.34 (*s*, 1H). FT–IR (ATR): ν (cm^−1^) = 3135 (*m*), 3109 (*m*), 2811 (*m*), 2681 (*m*), 2492 (*w*), 1618 (*w*), 1509 (*s*), 1472 (*m*), 1459 (*m*), 1396 (*m*), 1342 (*w*), 1279 (*m*), 1229 (*m*), 1214 (*s*), 1107 (*m*), 1062 (*s*), 1036 (*sh*), 923 (*m*), 870 (*w*), 815 (*m*) 759 (*m*), 723 (*s*), 654 (*m*), 623 (*m*), and 439 (*w*), m.p. 328–330. A clear, colorless plate-like crystal of C_4_H_6_N_2_O was grown from a dioxane reaction mixture slurry and mounted on MiTeGen loop with Parabar oil.

## Refinement

Crystal data, data collection and structure refinement details are summarized in Table 2 ▸. All non-hydrogen atoms were refined anisotropically. H atoms attached to carbon and oxygen were positioned geometrically and constrained to ride on their parent atoms. *U*_iso_(H) values were set to a multiple of *U*_eq_(C) with 1.2 for CH and 1.5 *U*_eq_(OH) for OH.

## Supplementary Material

Crystal structure: contains datablock(s) I. DOI: 10.1107/S2056989022002614/pk2662sup1.cif

Structure factors: contains datablock(s) I. DOI: 10.1107/S2056989022002614/pk2662Isup2.hkl

Supporting information file. DOI: 10.1107/S2056989022002614/pk2662Isup3.cml

CCDC reference: 2157074

Additional supporting information:  crystallographic information; 3D view; checkCIF report

## Figures and Tables

**Figure 4:**
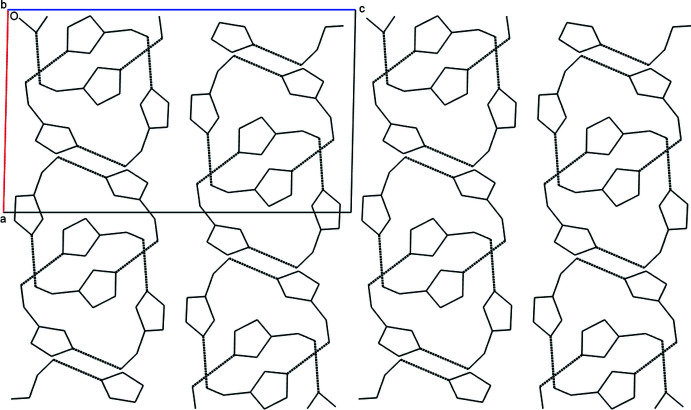
The packing arrangement of the three-mol­ecule supermolecule with hydrogen bonds represented as dashed lines as viewed along the *b* axis of the unit cell. The offset stacking of the three-membered supermolecule is due to the twofold screw axis orthogonal to this view.

**Table 1:** Hydrogen-bond geometry (Å, °)

*D*—H⋯*A*	*D*—H	H⋯*A*	*D*⋯*A*	*D*—H⋯*A*
O1—H1⋯N4	0.82	1.93	2.741 (2)	171
O2—H2*A*⋯N6	0.82	1.94	2.753 (2)	175
O3—H3*A*⋯N2	0.91 (3)	1.81 (3)	2.715 (2)	174 (2)

**Table 2:** Experimental details

Crystal data	
Chemical formula	C_4_H_6_N_2_O
*M*_r_	98.11
Crystal system, space group	Monoclinic, *P*2_1_/*n*
Temperature (K)	293
*a*, *b*, *c* (Å)	10.6201 (8), 8.0201 (8), 18.2085 (16)
β (°)	91.262 (7)
*V* (Å^3^)	1550.5 (2)
*Z*	12
Radiation type	Mo *K*α
μ (mm^−1^)	0.09
Crystal size (mm)	0.39 × 0.20 × 0.13

Data collection	
Diffractometer	XtaLAB Mini II
Absorption correction	Analytical [multi-faceted crystal analytical numeric absorption correction (Clark & Reid, 1995 ▸) and spherical harmonic empirical (using intensity measurements) absorption correction implemented in SCALE3 ABSPACK scaling algorithm]
*T*_min_, *T*_max_	0.876, 1.000
No. of measured, independent and observed [*I* > 2σ(*I*)] reflections	10521, 2764, 1722
*R*_int_	0.026
(sin θ/λ)_max_ (Å^−1^)	0.597

Refinement	
*R*[*F* ^2^ > 2σ(*F* ^2^)], *wR*(*F* ^2^), *S*	0.041, 0.110, 1.02
No. of reflections	2764
No. of parameters	197
No. of restraints	3
H-atom treatment	H atoms treated by a mixture of independent and constrained refinement
Δρ_max_, Δρ_min_ (e Å^−3^)	0.11, −0.13

Computer programs: *CrysAlis PRO* (Rigaku OD, 2021 ▸), *SHELXT2014/5* (Sheldrick, 2015*a* ▸), *SHELXL2014/7* (Sheldrick, 2015*b* ▸), and *OLEX2* (Dolomanov *et al.*, 2009 ▸).

## supplementary crystallographic information

### Crystal data

**Table:**

C_4_H_6_N_2_O	*D*_x_ = 1.261 Mg m^−^^3^
*M**_r_* = 98.11	Melting point: 328 K
Monoclinic, *P*2_1_/*n*	Mo *K*α radiation, λ = 0.71073 Å
*a* = 10.6201 (8) Å	Cell parameters from 945 reflections
*b* = 8.0201 (8) Å	θ = 2.2–22.5°
*c* = 18.2085 (16) Å	µ = 0.09 mm^−^^1^
β = 91.262 (7)°	*T* = 293 K
*V* = 1550.5 (2) Å^3^	Plate, colourless
*Z* = 12	0.39 × 0.20 × 0.13 mm
*F*(000) = 624	

### Data collection

**Table:**

XtaLAB Mini II diffractometer	2764 independent reflections
Radiation source: fine-focus sealed X-ray tube, Rigaku (Mo) X-ray Source	1722 reflections with *I* > 2σ(*I*)
Graphite monochromator	*R*_int_ = 0.026
Detector resolution: 10.0000 pixels mm^-1^	θ_max_ = 25.1°, θ_min_ = 2.2°
ω scans	*h* = −12→12
Absorption correction: analytical [multi-faceted crystal analytical numeric absorption correction (Clark & Reid, 1995) and spherical harmonic empirical (using intensity measurements) absorption correction implemented in SCALE3 ABSPACK scaling algorithm]	*k* = −9→9
*T*_min_ = 0.876, *T*_max_ = 1.000	*l* = −21→20
10521 measured reflections	

### Refinement

**Table:**

Refinement on *F*^2^	Hydrogen site location: mixed
Least-squares matrix: full	H atoms treated by a mixture of independent and constrained refinement
*R*[*F*^2^ > 2σ(*F*^2^)] = 0.041	*w* = 1/[σ^2^(*F*_o_^2^) + (0.0507*P*)^2^ + 0.1174*P*] where *P* = (*F*_o_^2^ + 2*F*_c_^2^)/3
*wR*(*F*^2^) = 0.110	(Δ/σ)_max_ < 0.001
*S* = 1.02	Δρ_max_ = 0.11 e Å^−^^3^
2764 reflections	Δρ_min_ = −0.13 e Å^−^^3^
197 parameters	Extinction correction: SHELXL2014/7 (Sheldrick 2015b), Fc^*^=kFc[1+0.001xFc^2^λ^3^/sin(2θ)]^-1/4^
3 restraints	Extinction coefficient: 0.0103 (17)
Primary atom site location: dual	

### Special details

**Table:**

Experimental. A clear, colorless platelike crystal of C_4_H_6_N_2_O was grown from a dioxane reaction mixture slurry. The crystal of dimensions 0.39 x 0.20 x 0.13 mm was mounted on MiTeGen loop with Parabar oil and diffraction data was collected. The asymmetric unit includes three units of the C_4_H_6_N_2_O molecule. The three crystallographically unique molecules are linked through hydrogen bonding in a head-to-tail fashion creating the pseudo-superstructure (C_4_H_6_N_2_O)_3_. Diffraction data was collected with a Rigaku XtaLAB Mini II benchtop X-ray diffractometer with a fine-focus sealed Mo-target X-ray tube (λ = 0.71073 Å) operated at 600 W power (50 kV, 12 mA) and a HyPix-Bantam Hybrid Photon Counting (HPC) Detector. The X-ray intensities were measured at 293 (2) K; the detector was placed at a distance 4.50 cm from the crystal. The collected frames were integrated with the CrysAlisPro 1.171.41.103a (Rigaku Oxford Diffraction, 2020) software package using a narrow-frame algorithm. Data were corrected for absorption effects using a multifaceted crystal analytical numeric absorption correction (Clark & Reid, 1995) and spherical harmonic empirical absorption correction implemented in the SCALE3 ABSPACK scaling algorithm. The space group was assigned using the GRAL algorithm within the CrysAlisPro 1.171.41.103a software package, solved with ShelXT (Sheldrick, 2015a) and refined with ShelXL (Sheldrick, 2015b) and the graphical interface Olex2 v1.3 (Dolomanov *et al.*, 2009).
Geometry. All esds (except the esd in the dihedral angle between two l.s. planes) are estimated using the full covariance matrix. The cell esds are taken into account individually in the estimation of esds in distances, angles and torsion angles; correlations between esds in cell parameters are only used when they are defined by crystal symmetry. An approximate (isotropic) treatment of cell esds is used for estimating esds involving l.s. planes.

### Fractional atomic coordinates and isotropic or equivalent isotropic displacement parameters (Å^2^)

**Table:**

	*x*	*y*	*z*	*U*_iso_*/*U*_eq_	
O1	0.77310 (12)	0.4000 (2)	0.34750 (8)	0.0820 (5)	
H1	0.7375	0.3864	0.3075	0.123*	
O3	0.13306 (15)	0.3271 (2)	0.40368 (8)	0.0817 (5)	
O2	0.35971 (13)	0.3114 (2)	0.05475 (7)	0.0858 (5)	
H2A	0.3164	0.3045	0.0914	0.129*	
N1	0.58494 (13)	0.40154 (18)	0.41424 (8)	0.0586 (4)	
N5	0.11264 (13)	0.25149 (18)	0.27851 (8)	0.0561 (4)	
N3	0.56345 (14)	0.29933 (19)	0.10954 (9)	0.0623 (4)	
N6	0.20362 (14)	0.2999 (2)	0.17339 (9)	0.0685 (5)	
N2	0.38798 (15)	0.3171 (2)	0.41771 (10)	0.0719 (5)	
N4	0.67175 (16)	0.3827 (2)	0.20794 (9)	0.0797 (5)	
C10	0.21491 (16)	0.2301 (2)	0.23800 (11)	0.0639 (5)	
H10	0.2861	0.1721	0.2541	0.077*	
C12	0.02984 (17)	0.3416 (2)	0.23675 (11)	0.0652 (5)	
H12	−0.0501	0.3765	0.2498	0.078*	
C8	0.64563 (18)	0.4199 (2)	0.08737 (12)	0.0683 (6)	
H8	0.6546	0.4598	0.0398	0.082*	
C11	0.08636 (19)	0.3702 (3)	0.17301 (11)	0.0698 (6)	
H11	0.0508	0.4296	0.1339	0.084*	
C2	0.46790 (19)	0.4180 (3)	0.38673 (11)	0.0713 (6)	
H2	0.4457	0.4928	0.3496	0.086*	
C4	0.57914 (19)	0.2808 (3)	0.46627 (11)	0.0715 (6)	
H4	0.6458	0.2406	0.4952	0.086*	
C6	0.5837 (2)	0.2836 (3)	0.18242 (12)	0.0735 (6)	
H6	0.5395	0.2097	0.2116	0.088*	
C3	0.4592 (2)	0.2309 (3)	0.46797 (11)	0.0724 (6)	
H3	0.4287	0.1490	0.4990	0.087*	
C7	0.71035 (19)	0.4689 (3)	0.14715 (14)	0.0752 (6)	
H7	0.7729	0.5501	0.1477	0.090*	
C1	0.69704 (19)	0.4933 (3)	0.39245 (13)	0.0814 (6)	
H1A	0.6713	0.5942	0.3668	0.098*	
H1B	0.7449	0.5258	0.4361	0.098*	
C9	0.0979 (2)	0.2020 (3)	0.35501 (12)	0.0816 (7)	
H9A	0.0106	0.1727	0.3628	0.098*	
H9B	0.1488	0.1038	0.3650	0.098*	
C5	0.4684 (2)	0.2148 (3)	0.06467 (13)	0.0879 (7)	
H5A	0.5030	0.1893	0.0171	0.105*	
H5B	0.4464	0.1103	0.0879	0.105*	
H3A	0.218 (2)	0.332 (3)	0.4093 (12)	0.102 (8)*	

### Atomic displacement parameters (Å^2^)

**Table:**

	*U*^11^	*U*^22^	*U*^33^	*U*^12^	*U*^13^	*U*^23^
O1	0.0559 (9)	0.1021 (11)	0.0885 (10)	0.0078 (8)	0.0108 (7)	0.0001 (9)
O3	0.0564 (10)	0.1227 (13)	0.0662 (9)	0.0023 (8)	0.0103 (7)	−0.0077 (9)
O2	0.0682 (10)	0.1331 (13)	0.0563 (8)	−0.0164 (9)	0.0034 (7)	0.0034 (9)
N1	0.0498 (9)	0.0594 (9)	0.0665 (10)	0.0011 (8)	−0.0004 (7)	−0.0084 (9)
N5	0.0466 (9)	0.0645 (10)	0.0574 (9)	−0.0078 (7)	0.0063 (7)	0.0055 (8)
N3	0.0534 (9)	0.0638 (10)	0.0702 (11)	0.0037 (8)	0.0149 (8)	−0.0010 (9)
N6	0.0578 (10)	0.0845 (12)	0.0635 (11)	−0.0012 (9)	0.0095 (8)	0.0008 (9)
N2	0.0554 (10)	0.0796 (11)	0.0805 (12)	−0.0047 (9)	−0.0062 (9)	−0.0010 (10)
N4	0.0668 (11)	0.0933 (13)	0.0791 (12)	0.0025 (9)	0.0057 (9)	0.0062 (11)
C10	0.0457 (11)	0.0722 (13)	0.0740 (13)	0.0037 (10)	0.0041 (9)	0.0058 (11)
C12	0.0457 (11)	0.0773 (13)	0.0725 (13)	0.0078 (10)	0.0027 (10)	−0.0010 (11)
C8	0.0551 (12)	0.0723 (14)	0.0783 (14)	0.0049 (11)	0.0191 (11)	0.0139 (12)
C11	0.0673 (13)	0.0761 (14)	0.0655 (13)	0.0034 (11)	−0.0071 (10)	0.0048 (11)
C2	0.0624 (13)	0.0704 (13)	0.0807 (14)	0.0078 (11)	−0.0082 (11)	0.0103 (12)
C4	0.0604 (13)	0.0911 (16)	0.0625 (12)	0.0091 (11)	−0.0090 (9)	0.0065 (12)
C6	0.0705 (13)	0.0794 (14)	0.0714 (14)	0.0028 (10)	0.0175 (10)	0.0156 (11)
C3	0.0729 (14)	0.0784 (14)	0.0658 (13)	−0.0035 (12)	0.0037 (10)	0.0086 (11)
C7	0.0572 (12)	0.0699 (14)	0.0988 (17)	0.0004 (11)	0.0065 (12)	0.0073 (13)
C1	0.0642 (13)	0.0802 (14)	0.1004 (16)	−0.0145 (12)	0.0140 (11)	−0.0131 (13)
C9	0.0713 (14)	0.1005 (17)	0.0732 (14)	−0.0241 (12)	0.0082 (11)	0.0170 (14)
C5	0.0811 (16)	0.0994 (17)	0.0840 (15)	−0.0118 (14)	0.0237 (12)	−0.0260 (14)

### Geometric parameters (Å, º)

**Table:**

O1—H1	0.8200	N4—C7	1.375 (2)
O1—C1	1.383 (2)	C10—H10	0.9300
O3—C9	1.384 (3)	C12—H12	0.9300
O3—H3A	0.91 (3)	C12—C11	1.338 (2)
O2—H2A	0.8200	C8—H8	0.9300
O2—C5	1.399 (3)	C8—C7	1.334 (3)
N1—C2	1.336 (2)	C11—H11	0.9300
N1—C4	1.357 (2)	C2—H2	0.9300
N1—C1	1.462 (2)	C4—H4	0.9300
N5—C10	1.337 (2)	C4—C3	1.336 (3)
N5—C12	1.358 (2)	C6—H6	0.9300
N5—C9	1.460 (2)	C3—H3	0.9300
N3—C8	1.369 (2)	C7—H7	0.9300
N3—C6	1.346 (2)	C1—H1A	0.9700
N3—C5	1.452 (3)	C1—H1B	0.9700
N6—C10	1.306 (2)	C9—H9A	0.9700
N6—C11	1.367 (2)	C9—H9B	0.9700
N2—C2	1.309 (2)	C5—H5A	0.9700
N2—C3	1.363 (2)	C5—H5B	0.9700
N4—C6	1.305 (3)		
			
C1—O1—H1	109.5	N1—C4—H4	126.6
C9—O3—H3A	110.9 (15)	C3—C4—N1	106.74 (17)
C5—O2—H2A	109.5	C3—C4—H4	126.6
C2—N1—C4	106.01 (16)	N3—C6—H6	123.5
C2—N1—C1	127.22 (18)	N4—C6—N3	113.06 (18)
C4—N1—C1	126.76 (17)	N4—C6—H6	123.5
C10—N5—C12	106.44 (15)	N2—C3—H3	124.8
C10—N5—C9	126.65 (17)	C4—C3—N2	110.35 (19)
C12—N5—C9	126.66 (16)	C4—C3—H3	124.8
C8—N3—C5	127.10 (18)	N4—C7—H7	124.7
C6—N3—C8	105.55 (17)	C8—C7—N4	110.6 (2)
C6—N3—C5	127.22 (17)	C8—C7—H7	124.7
C10—N6—C11	104.29 (15)	O1—C1—N1	112.21 (16)
C2—N2—C3	104.31 (17)	O1—C1—H1A	109.2
C6—N4—C7	104.10 (18)	O1—C1—H1B	109.2
N5—C10—H10	123.8	N1—C1—H1A	109.2
N6—C10—N5	112.49 (17)	N1—C1—H1B	109.2
N6—C10—H10	123.8	H1A—C1—H1B	107.9
N5—C12—H12	126.9	O3—C9—N5	112.38 (16)
C11—C12—N5	106.23 (17)	O3—C9—H9A	109.1
C11—C12—H12	126.9	O3—C9—H9B	109.1
N3—C8—H8	126.6	N5—C9—H9A	109.1
C7—C8—N3	106.73 (18)	N5—C9—H9B	109.1
C7—C8—H8	126.6	H9A—C9—H9B	107.9
N6—C11—H11	124.7	O2—C5—N3	112.05 (17)
C12—C11—N6	110.54 (18)	O2—C5—H5A	109.2
C12—C11—H11	124.7	O2—C5—H5B	109.2
N1—C2—H2	123.7	N3—C5—H5A	109.2
N2—C2—N1	112.59 (18)	N3—C5—H5B	109.2
N2—C2—H2	123.7	H5A—C5—H5B	107.9
			
N1—C4—C3—N2	0.2 (2)	C4—N1—C2—N2	0.3 (2)
N5—C12—C11—N6	0.0 (2)	C4—N1—C1—O1	77.3 (2)
N3—C8—C7—N4	−0.1 (2)	C6—N3—C8—C7	0.3 (2)
C10—N5—C12—C11	0.0 (2)	C6—N3—C5—O2	95.1 (2)
C10—N5—C9—O3	90.6 (2)	C6—N4—C7—C8	−0.2 (2)
C10—N6—C11—C12	0.0 (2)	C3—N2—C2—N1	−0.2 (2)
C12—N5—C10—N6	0.0 (2)	C7—N4—C6—N3	0.3 (2)
C12—N5—C9—O3	−82.8 (2)	C1—N1—C2—N2	179.75 (16)
C8—N3—C6—N4	−0.4 (2)	C1—N1—C4—C3	−179.76 (17)
C8—N3—C5—O2	−80.2 (2)	C9—N5—C10—N6	−174.54 (17)
C11—N6—C10—N5	0.0 (2)	C9—N5—C12—C11	174.51 (18)
C2—N1—C4—C3	−0.3 (2)	C5—N3—C8—C7	176.39 (18)
C2—N1—C1—O1	−102.0 (2)	C5—N3—C6—N4	−176.51 (19)
C2—N2—C3—C4	0.0 (2)		

### Hydrogen-bond geometry (Å, º)

**Table:**

*D*—H···*A*	*D*—H	H···*A*	*D*···*A*	*D*—H···*A*
O1—H1···N4	0.82	1.93	2.741 (2)	171
O2—H2*A*···N6	0.82	1.94	2.753 (2)	175
O3—H3*A*···N2	0.91 (3)	1.81 (3)	2.715 (2)	174 (2)
